# Nitrofurazone Removal from Water Enhanced by Coupling Photocatalysis and Biodegradation

**DOI:** 10.3390/ijms22042186

**Published:** 2021-02-22

**Authors:** Wojciech Smułek, Zuzanna Bielan, Amanda Pacholak, Agata Zdarta, Agnieszka Zgoła-Grześkowiak, Anna Zielińska-Jurek, Ewa Kaczorek

**Affiliations:** 1 Institute of Chemical Technology and Engineering, Poznan University of Technology, Berdychowo 4, 60-965 Poznan, Poland; amanda.d.pacholak@doctorate.put.poznan.pl (A.P.); agata.zdarta@put.poznan.pl (A.Z.); 2 Department of Process Engineering and Chemical Technology, Gdansk University of Technology, Gabriela Narutowicza 11/12, 80-233 Gdansk, Poland; bielan_chan@onet.eu (Z.B.); annjurek@pg.edu.pl (A.Z.-J.); 3 Institute of Chemistry and Technical Electrochemistry, Poznan University of Technology, Berdychowo 4, 60-965 Poznan, Poland; agnieszka.zgola-grzeskowiak@put.poznan.pl

**Keywords:** photocatalysis, biodegradation, nitrofurazone, photodegradation, pharmaceuticals, TiO_2_, wastewater

## Abstract

(1) Background: Environmental contamination with antibiotics is particularly serious because the usual methods used in wastewater treatment plants turn out to be insufficient or ineffective. An interesting idea is to support natural biodegradation processes with physicochemical methods as well as with bioaugmentation with efficient microbial degraders. Hence, the aim of our study is evaluation of the effectiveness of different methods of nitrofurazone (NFZ) degradation: photolysis and photodegradation in the presence of two photocatalysts, the commercial TiO_2_-P25 and a self-obtained Fe_3_O_4_@SiO_2_/TiO_2_ magnetic photocatalyst. (2) Methods: The chemical nature of the photocatalysis products was investigated using a spectrometric method, and then, they were subjected to biodegradation using the strain *Achromobacter xylosoxidans* NFZ2. Additionally, the effects of the photodegradation products on bacterial cell surface properties and membranes were studied. (3) Results: Photocatalysis with TiO_2_-P25 allowed reduction of NFZ by over 90%, demonstrating that this method is twice as effective as photolysis alone. Moreover, the bacterial strain used proved to be effective in the removal of NFZ, as well as its intermediates. (4) Conclusions: The results indicated that photocatalysis alone or coupled with biodegradation with the strain *A. xylosoxidans* NFZ2 leads to efficient degradation and almost complete mineralization of NFZ.

## 1. Introduction

Increasing production and consumption of medicines has led to increased environmental pollution with pharmaceuticals [1]. Of particular concern is the uncontrolled use of over-the-counter (OTC) drugs. Not only the scale of their use but also their disposal pose a significant threat [2,3]. These drugs get into the environment from municipal sewage or are washed away by rainwater and groundwater from landfills [4,5,6]. An additional source of environmental pollution with pharmaceuticals is industrial animal farms, where antibiotics are an important feed additive [7,8]. This group of compounds includes nitrofuran derivatives, like nitrofurazone [9]. Nitrofurazone belongs to the compounds showing bactericidal activity against both Gram-negative and Gram-positive bacterial cells. Nitrofurazone (NFZ) is used to treat infections in humans as well as domestic and farm animals. It is primarily used in preparations applied externally on the skin [10]. Due to the relatively high toxicity and possible carcinogenicity, this compound has been excluded from use in the United States and European Union (EU) countries. However, even in those countries, its illegal use is still noted [11]. Importantly, the prohibition on nitrofurazone in EU countries only applies to its use in animal breeding [12].

So far, studies focused on nitrofurazone degradation and its removal from the environment are severely limited. They mostly come from experiments carried out by the groups of Hou et al. [13,14], Kong et al. [15,16], as well as De Luca et al. [17]. Hou et al. examined the degradation of NFZ using the bio-photoelectrochemical system, which was a combination of photocatalysis with the microbial electrochemical process. Apart from enhanced NFZ degradation [13], they achieved spontaneous electricity generation [14]. Their experiment reached an NFZ removal of 80–90% in 12 h, depending on the initial concentration used [13]. Kong et al. [16] focused on bio-electrodegradation. In a 6-hour experiment, they obtained almost complete degradation of NFZ starting from the initial concentration of 50 mg L^−1^. Moreover, they observed significant changes in the microbial community of biofilm after electrical stimulation. The results of the experiments in question have undoubtedly demonstrated the particular usefulness of the combination of biological (e.g., bio-electrodegradation) and physicochemical processes (like photocatalysis) in the removal of antibiotics like NFZ. 

It should also be noted that standard methods of wastewater treatment are mostly ineffective toward pharmaceuticals [18,19]. Moreover, the presence of medicines can disturb the functioning of activated sludge and be toxic to the microorganisms present in it. In light of the above, one of the most important issues related to the removal of pharmaceuticals is the development of new and effective strategies for their degradation, including that of nitrofurazone. Recently, photolytic and photocatalytic degradation methods are recognized as more efficient in the removal of pharmaceuticals from wastewater [20]. In the photocatalytic process, free-radical species are produced in situ, e.g., hydroxyl radical (HO), superoxide anion radical (O_2_¯), hydroperoxyl radical (HO_2_), alkoxyl radical (RO), or single oxygen (^1^O_2_), which can initiate the degradation of active pharmaceutical ingredients (APIs) to less toxic molecules more susceptible to biological degradation [21]. Titanium (IV) oxide is the most commonly used photocatalyst for environmental applications. However, in a technological process, there are challenges with photocatalytic efficiency and reusability, owing to its nanometric particle size [22]. In this study, TiO_2_ nanoparticles were immobilized on magnetite (Fe_3_O_4_) to improve photocatalyst separation. The developed surface area of the TiO_2_–SiO_2_ photocatalyst layer improved the direct contact between reactive oxygen species and organic molecules in-situ-generated on the photocatalyst surface. The combined photocatalytic and biological treatment enabled the degradation of emerging contaminants not susceptible to only biodegradation, whose photocatalytic mineralization at the same time would be not economically reasonable. 

Given the low efficiency of biodegradation, our objective was to assess the possibilities of supporting biodegradation with photocatalytic processes in NFZ removal. A significant novelty of our research was the development of the photocatalytic–biological coupled system for NFZ degradation. In this study, for the first time, a promising approach to removing NFZ and nitrofuran derivatives was reported. 

## 2. Results

### 2.1. Nitrofurazone Photodegradation

**Figure 1:**
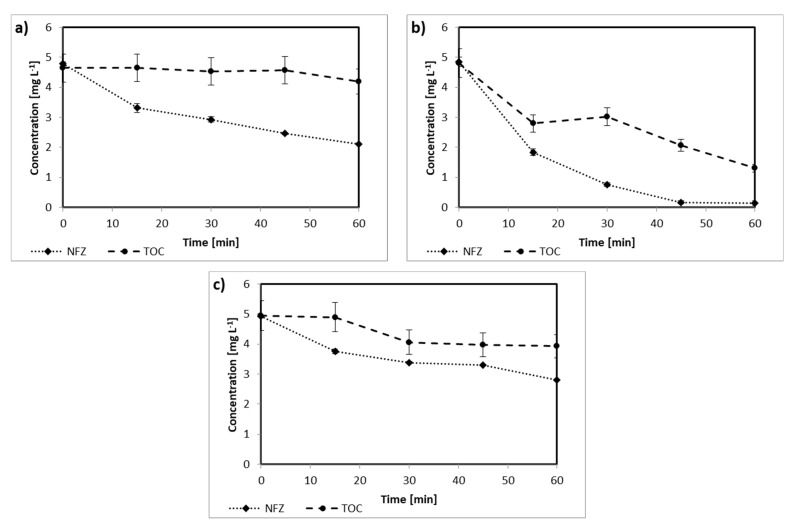
Degradation of nitrofurazone (NFZ) and changes in total organic carbon (TOC) concentration: (**a**) photodegradation without a photocatalyst, (**b**) photocatalytic degradation with TiO_2_-P25, and (**c**) photocatalytic degradation in the presence of Fe_3_O_4_@SiO_2_/TiO_2_.

The first stage of the study focused on the photodegradation of NFZ. The experiments allowed us to analyze both changes in the concentration of NFZ and the mineralization efficiency measure as total organic carbon (TOC) removal. The photolysis led to a 50% reduction in the NFZ concentration in the samples after 60 min of the process (Figure 1a). However, a very low decrease in the TOC concentration was observed (down to 4.2 mg L^−1^), suggesting transformation but not mineralization of the contaminants. 

When TiO_2_-P25 was applied as a photocatalyst, the efficiency of degradation significantly increased (see Figure 1b). For this sample, not only almost complete removal of NFZ was observed, but also the content of TOC significantly changed; after 30 min, it was 3.0 mg L^−1^, while after 60 min, it did not exceed 1.3 mg L^−1^. As presented in Figure 1c, the concentration of nitrofurazone in the presence of Fe_3_O_4_@SiO_2_/TiO_2_ containing less amount of TiO_2_ in the structure when compared to pure TiO_2_-P25 decreased to 2.8 mg L^−1^ after 60 min of irradiation. The TOC decay was relatively less, and after 30 min, it was only 20%, and this value did not change considerably during further processing. However, application of the magnetic photocatalyst Fe_3_O_4_@SiO_2_/TiO_2_ facilitated the photocatalyst separation after the photodegradation process. 

**Figure 2:**
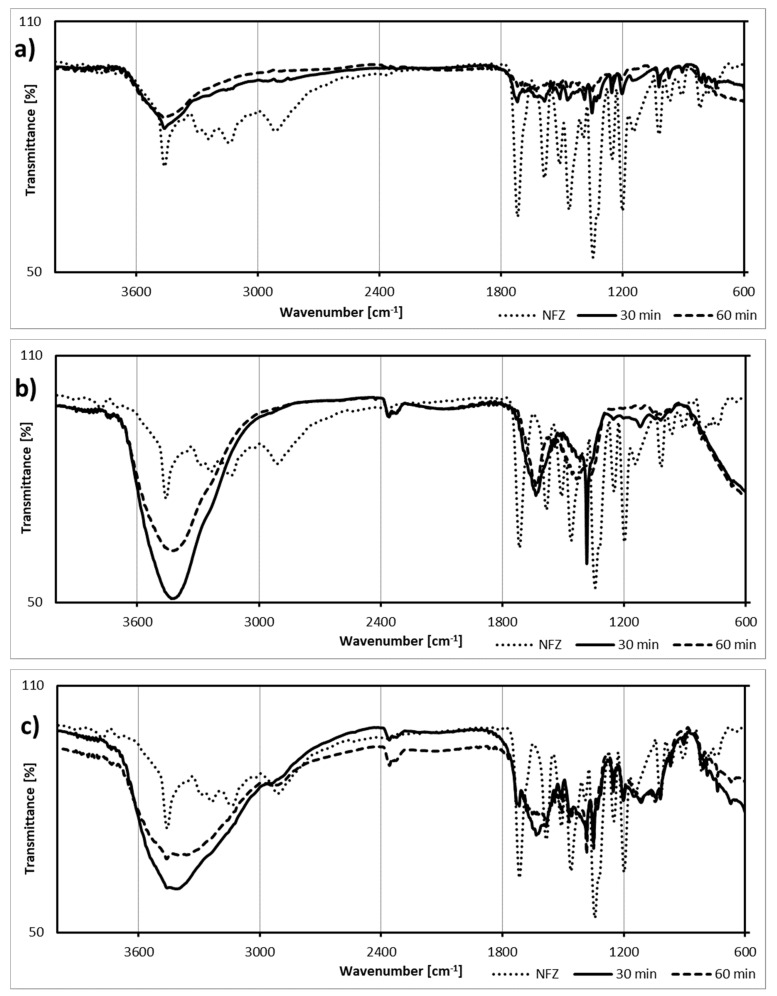
Infrared spectra of nitrofurazone (NFZ) and its degradation products: (**a**) photodegradation without a catalyst, (**b**) photocatalytic degradation with TiO_2_-P25, and (**c**) photocatalytic degradation with Fe_3_O_4_@SiO_2_/TiO_2_.

Additional information was obtained by analysis of the infrared spectra of the degradation products (Figure 2). The initial spectrum of NFZ contained intensive signals assigned to the N-H bonds in secondary amines (3450 cm^−1^) and to the C-H bonds attached at the furan ring (ca. 3100 cm^−1^). Moreover, the spectrum showed signals from the C-O bond, as well as the bonds in the nitro group (1330 cm^−1^). Additionally, the band at 1720 cm^−1^ confirmed the presence of a carbonyl group.

The spectra of the degradation products showed detectable signals assigned to O-H bonds (3400 cm^−1^), especially intensive and broad in the case of products from photocatalytic processes (Figure 2b,c). What is more, the signal at 1630 cm^−1^ may indicate the presence of a furan ring. Nevertheless, the decay of the 2900 cm^−1^ signal, observed in the spectra of the samples after degradation, may indicate the fission of the side chain and vanishing of the C-H group. 

### 2.2. Nitrofurazone Biodegradation

**Figure 3:**
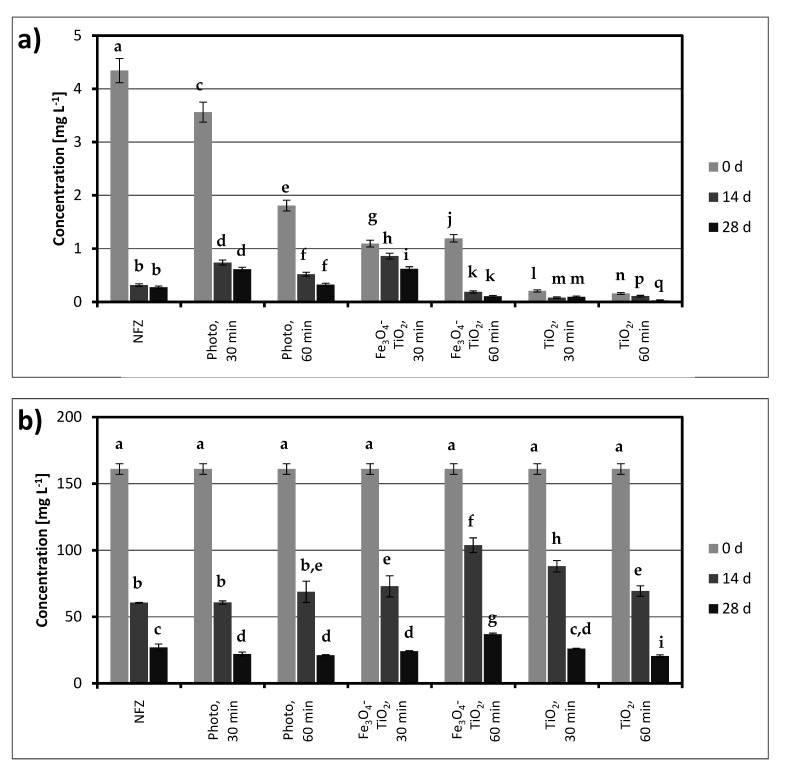
Biological degradation of nitrofurazone (NFZ) and photodegradation products: (**a**) NFZ concentration and (**b**) total organic carbon (TOC) concentration (photo, photodegradation without a catalyst; TiO_2_-P25, photocatalytic degradation with P25; Fe_3_O_4_-TiO_2_, photocatalytic degradation with Fe_3_O_4_@SiO_2_/TiO_2_); the values, which are not statistically different in data series, are marked with the same lowercase letters (“a”, “b”, etc.).

The next step was to perform degradation experiments using the *A. xylosoxidans* NFZ2 strain. The obtained results are presented in Figure 3. In a system containing initially only NFZ, biodegradation was close to 90% in 14 days, but after 28 days, it did not change significantly. Noticeably higher NFZ concentrations (at the end of the experiment) were found for the systems after photodegradation and photocatalytic degradation in the presence of Fe_3_O_4_@SiO_2_/TiO_2_. The most effective biodegradation occurred in the cultures after photocatalysis with TiO_2_-P25, but a relatively low concentration of NFZ at the beginning of biodegradation played an important role here.

Similar conclusions can be drawn based on the measurement of TOC changes (Figure 3b). The high initial values of this parameter are due to the addition of sodium succinate at the beginning of the biodegradation. Preliminary biodegradation studies showed that in cultures with no other readily available carbon source added, there was no degradation. In addition, TOC changes showed that the lowest final concentrations (ca. 20 mg L^−1^) were observed for the system after 60 min of photocatalysis with TiO_2_-P25.

### 2.3. Effect of Nitrofurazone and Its By-Products on Bacterial Cells

**Figure 4:**
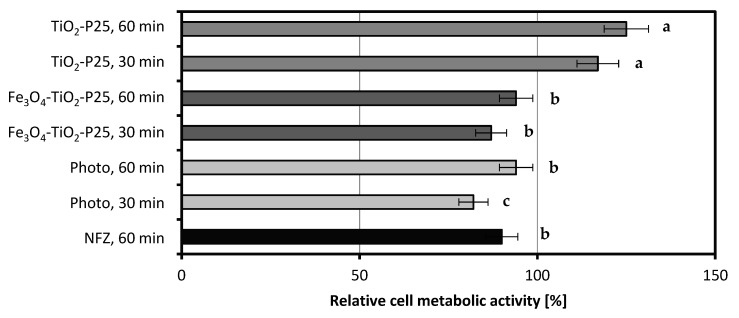
Relative cell metabolic activity after exposure to nitrofurazone and its degradation products; the value of 100% was assumed for cells exposed to sodium succinate (photo, photodegradation without a catalyst; TiO_2_-P25, photocatalytic degradation with P25; Fe_3_O_4_-TiO_2_, photocatalytic degradation with Fe_3_O_4_@SiO_2_/TiO_2_); the values, which are not statistically different in data series, are marked with the same lowercase letters (“a”, “b”, etc.).

Figure 4 presents the results of bacterial cell metabolic activity tested in the presence of NFZ and its degradation products obtained as a result of catalytic and photocatalytic processes. The metabolic activity was measured after 3 days of incubation of the bacteria with a sterile solution of NFZ or liquids obtained after 30 min and 60 min photolysis as well as photocatalysis (with two photocatalysts separately: Fe_3_O_4_@SiO_2_/TiO_2_ and TiO_2_-P25). 

The cell metabolic activity of the strain *A. xylosoxidans* NFZ2 in the presence of NFZ was measured as 90% of that of the control sample. The products of 30 min photolytic reaction resulted in a decrease in the cell metabolic activity of bacteria to 82%; however, in the presence of the products of the 60 min reaction, an increase in the bacterial cell metabolic activity to 94% was observed. Referring to the degradation test (Figure 1a), we can assume that the occurring transformation led to the formation of products even more toxic than NFZ itself. The application of photocatalysts, especially TiO_2_-P25, resulted in the formation of less toxic degradation products. The bacteria that were exposed to nitrofurazone’s degradation products were characterized by lower cell metabolic activity in the 30 min processes (87% for Fe_3_O_4_@SiO_2_/TiO_2_ and 117% for TiO_2_-P25) than in the 60 min ones (94% for Fe_3_O_4_@SiO_2_/TiO_2_ and 125% for TiO_2_-P25). 

To conclude, the products obtained as a result of longer photo(cata)lytic degradation (60 min) showed reduced toxicity toward the applied bacterial strain than the ones obtained from the 30 min reactions in all setups tested (photolysis as well as photocatalysis with catalysts Fe_3_O_4_@SiO_2_/TiO_2_ and TiO_2_-P25). Nevertheless, the catalyst TiO_2_-P25 allowed the decomposition of NFZ to less toxic products than the photocatalyst Fe_3_O_4_@SiO_2_/TiO_2_ did. 

Both the toxicity of the compound and the effectiveness of its biodegradation are strongly dependent on its bioavailability to the cells of microorganisms. Analyzing the changes in *A. xylosoxidans* NFZ2 cell surface properties listed in Table 1, it can be concluded that the photodegradation products modify the cell membrane permeability more strongly than nitrofurazone alone. 

As a result of contact with the mixture of compounds left after photocatalytic degradation, the cell membrane became practically impermeable, which may indicate a defensive reaction of the cells against the toxic effects of these compounds. Much less significant changes occurred after contact with the solution left after photolysis without the participation of a photocatalyst. Trends in the changes in cell adsorption properties were similar to those in the cell membrane permeability, although not as significant. Compared to the control sample or cells exposed to NFZ, for which the Congo red adsorption was 19 ± 6% and 17 ± 2%, respectively, in samples with solutions left after photocatalytic degradation, the values of the indicator of adsorption oscillated around 10%.

## 3. Discussion

Photo(cata)lytic degradation methods attract the attention of scientists due to their great potential for application in many fields, such as coating technology, air pollution treatment, and environmental pollution removal [23]. Nonetheless, the generated toxic by-products are a significant drawback that must be overcome [24]. The hybridization of photocatalysis and biological degradation might be a long-searched solution, and that concept was investigated in our study. Attempts were made not only to get a better understanding of the degradation potential of photolytic and photocatalytic solutions but also to evaluate the toxicity of the reaction products to bacterial cells and their impact on their properties.

One of the very few and isolated studies on nitrofuran derivatives photolysis is that described by De Luca et al. [17]. The authors observed that the total decay of NFZ under UV radiation needed ca. 60 min for an initial concentration of 20 mg L^−1^ and over 180 min when the initial concentration was 100 mg L^−1^. However, to obtain such efficiency, a UV source of relatively high power (250 W) was applied. Szabó-Bárdos et al. [25] focused on nitrofurantoin photodegradation, and starting with a concentration of 10 mg L^−1^, they obtained nearly 100% degradation after 80 min. Nevertheless, in both mentioned papers, no information was given on total mineralization; thus only an estimation of the degradation effectiveness could have been made. As observed in the case of photolysis alone (Figure 1a), the disappearance of the initial compound is not tantamount to the simultaneous removal of the TOC. 

The presented infrared spectra highlight changes in signals at 1630 cm^−1^ (increase) and 2900 cm^−1^ (decrease). The increase in this signal intensity and a decrease in the intensity of the signal from carbonyl in the amide group corroborate the considerations of De Luca et al. [17] on NFZ photodegradation products. At first, the double bond configuration in the NFZ molecule changes (photoisomerization occurs), and these changes are followed by the shortening of the molecule’s chain at the furan ring. Assuming that the breakdown will be analogous to that of nitrofurantoin (described by [25]), the resulting compounds contain more hydroxyl groups than NFZ and practically no amino groups. Moreover, the furan ring is relatively stable and degrades at further stages of the process. On the other hand, Kong et al. [15], studying the cathodic degradation of NFZ, revealed the main intermediates as, among others, [(5-hydroxyamino-2-furyl)-methyl]-hydrazinecarboxamide, [(5-amino-2-furyl)-methylene]hydrazine-carboxamide, and 5-hydroxy-cadaverine and 5-aminopentanamide, i.e., compounds containing amino groups.

It is worth mentioning that so far, only a few studies have been undertaken on the biodegradation of NFZ by bacterial strains. Hence, our study can be considered one of the pioneering ones in this area. It should be noted, however, that Kong et al. [16] used bio-electrodegradation to remove NFZ. The authors used bacteria capable of nitroaromatic reduction and electron transfer (e.g., *Klebsiella*, *Enterococcus*, *Citrobacter*, and *Desulfovibrio*). However, the removal of nearly 95% of the initial amount of the pharmaceutical took over 3 h. Hou et al. [13] applied a bio-photoelectrochemical system for NFZ degradation with the bacteria community coming from the domestic wastewater system. Ne, the obtained results showed that the tested system reached an 80% efficiency in 24 h. It should be compared with the results of photocatalysis, which allows only 20% removal of NFZ. More efficient was the second solution described also by Hou et al. [14], who obtained nearly 90% degradation of NFZ in 12 h in an analogous system. However, it should be emphasized that both methods described by Hou et al. [13,14] are relatively complex, and thus, they could be hard to implement in a wastewater treatment plant. The photocatalysis followed by biological degradation proposed in this study would be much easier to control. 

In our study, the differences observed in bacterial metabolic activity between samples and the results of biological degradation and the total organic carbon measurements are coherent. The bacteria from the systems that showed the highest degradation efficiency also exhibited the highest metabolic activity. Bergheim et al. [26] studied, among others, the toxicity of some antibiotics on the cells of *P. putida*. Their results showed that UV treatment reduced the antimicrobial activity of the antibiotics tested and increased the viability of the bacteria. Similar observations were made in our study. Moreover, our previous research showed that the presence of another 5-nitrofuran derivative, nitrofurantoin, contributed to a decrease in the cell metabolic activity of newly isolated environmental bacterial strains [27]. 

The issues of bioavailability and changes in cell surface properties induced by contact with nitrofuran-derived antibiotics have not yet been analyzed in detail. We noticed in our previous research that contact with nitrofurantoin led to a decrease in Congo red adsorption on *Sphingomonas paucimobilis* and *Rhizobium radiobacter* [27]. However, in the case of *Sphingobacterium thalpophilum* cells, an increase in the tested parameter was noticed. It should be noted that the cells of all the above-mentioned strains showed lower membrane permeability in the presence of nitrofurantoin than in cultures without this active pharmaceutical ingredient. It is worth mentioning that an antibiotic from another group, polymyxin B, caused an increase in the cell membrane permeability of *E. coli* and *S. aureus*. Hence, the response of the cell membrane depends on the bacterial strain as well as on the specific antibiotic. Nevertheless, all collected and discussed results show that the bioavailability of pharmaceuticals is one of the crucial factors directly related to the toxicity of biodegraded compounds.

## 4. Materials and Methods

### 4.1. Chemicals and Materials

All chemicals used in the experiments, including nitrofurazone, were purchased from Sigma-Aldrich (Poznan, Poland) and were of the highest analytical purity grade. To prepare all media and aqueous solutions, ultra-purified Milli-Q water (Arium^®^ Pro, Sartorius, Kostrzyn Wlkp., Poland) was used. All the solutions and glassware were sterilized prior to use in the experiments. To prevent contamination, a laminar flow cabinet was used when handling biological samples.

### 4.2. Bacterial Strain

The used bacterial strain, which was proved capable of degrading NFZ, was isolated from samples collected aseptically from a wastewater treatment plant in Poznań, Poland, analogically as described by Pacholak et al. [27]. Genetic identification based on comparing the 16S RNA sequences allowed the determination of its taxonomic affiliation. The strain was submitted to GenBank of the NCBI database as *Achromobacter xylosoxidans* strain NFZ2 (accession No. MK493330.1). The bacterial strain was stored on nutrient agar plates at 4 °C and transferred to fresh plates every 21 days.

### 4.3. Photocatalytic Degradation

The possibility of nitrofurazone photodegradation was determined in a 1 L batch reactor with a centrally located 24 W UV-A lamp (OSRAM DULUX L BLUE UVA) upon magnetic stirring. Two photocatalysts were used: the first one was the commercially available TiO_2_-P25 (AEROXIDE, Evonik, Essen, Germany), and the second one, Fe_3_O_4_@SiO_2_/TiO_2_, was synthesized by us; the parameters characterizing them are given in Table 2. 

Fe_3_O_4_@SiO_2_/TiO_2_ was obtained by the water/oil microemulsion method to create a stable core (Fe_3_O_4_) and shell (TiO_2_) structure, with an inert silica interlayer between them. The detailed preparation procedure as well as physicochemical and photocatalysis characterization are given in Zielińska-Jurek et al. [28,29]. Such core–shell photocatalyst was chosen for nitrofurazone degradation because of its good stability and photoactivity presented in several research publications [21,28,29,30]. In comparison to TiO_2_-P25, whose particles are nano-sized and thus difficult to separate, Fe_3_O_4_@SiO_2_/TiO_2_ is easy to separate using an external magnetic field, without a decrease in its photoactivity. The mass of the active photocatalyst in used the magnetic composite (2 g) was 1.55 g. 

To study the photocatalytic degradation of nitrofurazone, 2 g L^−1^ of the photocatalyst (TiO_2_-P25 or Fe_3_O_4_@SiO_2_/TiO_2_) was dispersed in 1 L of a 5 mg L^−1^ solution of nitrofurazone and stirred for 30 min without irradiation to establish absorption–desorption equilibrium. After collecting the reference sample, the dispersion was irradiated for 1 h. Every 15 min, 1 mL of the suspension was collected, and the photocatalyst nanoparticles were separated from the suspension by filtration combined with an external magnetic field.

### 4.4. Biological Degradation

To obtain liquid cultures, inocula were prepared by adding a loopful of cells, taken from an agar plate, to the sterilized nutrient broth. After their incubation at 30 °C with shaking at 120 rpm (KS 4000 ic control, IKA Werke GmbH, Staufen, Germany) for 24 h, the biomass was centrifuged at 4000× *g* for 10 min (3–18K, Sigma Laborzentrifugen GmbH, Osterode am Harz, Germany) and washed twice with mineral salt medium. Then, the bacteria were re-suspended in a sterile medium to reach the final bacteria concentration of 1 × 10^9^ cfu mL^−1^ (in log phase; optical density 1.0 at *λ* = 600 nm; spectrophotometer Jasco V-650, Tokyo, Japan). The bacterial cultures used in the experiments contained 40 mL of nitrofurazone water solution (5 mg L^−1^) or 40 mL of the filtrated (0.2 μm) solution after photocatalytic degradation, 5 mL of bacteria inoculum, 0.1 mL of sodium succinate (20% aqueous solution), and 0.1 mL of trace element solution (concentration in g L^−1^: MgSO_4_∙7H_2_O, 0.35; FeSO_4_∙7H_2_O, 0.035; CuSO_4_∙7H_2_O, 0.2; MnSO_4_∙5H_2_O, 0.2; ZnCl_2_, 0.105; CoSO_4_∙7H_2_O, 0.025; and H_3_BO_3_, 0.285). Moreover, 5 mL of the concentrate of mineral salt medium was added to obtain final salt concentrations as follows: (in g L^−1^) Na_2_HPO_4_∙2H_2_O, 7.0; KH_2_PO_4_, 2.8; NaCl, 0.5; and NH_4_Cl, 1.0.

The bacterial cultures (biodegradation tests) were incubated for 28 days at 30 °C with shaking at 120 rpm. They were grown in sterile 250 mL Duran^®^ Schott (Wertheim, Germany) laboratory glass bottles. 

### 4.5. Chemical Analysis of Nitrofurazone Degradation

The nitrofurazone concentration in the post-photo(cata)lysis filtrate was preliminarily evaluated using a high-pressure liquid chromatograph (HPLC) equipped with a C18 chromatography column (Phenomenex, 150 × 4.6 mm) and a diode array detector (model SPD-M20A, Shimadzu, Kyoto, Japan). Detection was carried out at 372 nm using the external calibration method. The mobile phase was composed of 5 × 10^−3^ mol L^−1^ of ammonium acetate solution and methanol in a volume ratio 1:1 at a flow rate of 1 mL min^−1^. The injection volume was 5 µL, and the analysis temperature was maintained at 35 °C. 

The concentration of residual nitrofurazone was determined using an LC-MS/MS system comprising an UltiMate 3000 RSLC chromatograph from Dionex (Sunnyvale, CA, USA) and an API 4000 QTRAP triple quadrupole mass spectrometer from AB Sciex (Foster City, CA, USA). Samples of 5 µL were injected into a Kinetex Evo C18 column (150 mm × 2.1 mm I.D.; 2.6 µm) from Phenomenex (Torrance, CA, USA), maintained at 35 °C. The mobile phase used in the analysis consisted of 5 × 10^−3^ mol L^−1^ of ammonium acetate in water and methanol at a flow rate of 0.3 mL min^−1^. Gradient elution was performed by linearly increasing the percentage of organic modifier from 75% to 80% in 2 min and then from 80% to 100% in 1 min. A pre-run time of 3 min was used before the injection. The LC column effluent was directed to the electrospray ionization source (Turbo Ion Spray) of the mass spectrometer. The Turbo Ion Spray source operated in positive ion mode. The following settings were used for the ion source and mass spectrometer: curtain gas, 10 psi; nebulizer gas, 40 psi; auxiliary gas, 40 psi; temperature, 400 °C; ion spray voltage, 4500 V; declustering potential, 60 V; and collision gas set to medium. The dwell time for each mass transition detected in the selected reaction monitoring mode was set to 200 ms. The quantitative transition was from 199 to 182 m/z at collision energy set to 17 eV and collision cell exit potential set to 11 V. The confirmatory transition was from 199 to 156 m/z at collision energy set to 19 eV and collision cell exit potential set to 9 V. 

Additionally, the mineralization of the organic compounds in the samples was evaluated by measuring the total organic carbon (TOC) concentration (TOC Analyzer, Shimadzu, Kyoto, Japan). Moreover, the nitrofurazone photocatalytic degradation products were studied. The 50 mL solutions after degradation were freeze-dried for 48 h at −55 °C under a pressure of 0.37 mbar (freeze dryer Alpha 1-2 LD plus, Christ, Osterode, Germany). Then, 2 mg of the samples (cells or extracellular compounds) were mixed with 200 mg of anhydrous potassium bromide and compressed into a1 mm disc, and infrared spectra (from 600 cm^−1^ to 4000 cm^−1^) were recorded (Vertex 70, Bruker, Karlsruhe, Germany). 

### 4.6. Bacterial Metabolic Activity Assessment

To determine the toxicity of nitrofurazone and its photocatalytic degradation products, the cells’ metabolic activity was analyzed using 3-(4,5-dimethylthiazol-2-yl)-2,5-diphenyltetrazolium bromide assay (MTT) in accordance with the method described by Wang et al. [31]. The cultures were prepared according to the methodology described in Section 4.4. For the control sample, only sodium succinate was used. After 24 h of incubation, the microbial cultures were centrifuged (4000× *g*, 10 min), washed with mineral salt medium, and re-suspended in MSM to obtain OD600 equal to 0.1. Thereafter, 0.9 mL of the microbial suspension was incubated with 0.1 mL of 5 g L^−1^ MTT solution for 2 h at 30 °C. After incubation, the cultures were centrifuged at 15,000× *g*, the supernatant was removed, and the pellet (formazan precipitate formed by viable cells) was dissolved with 1 mL of propan-2-ol. Afterward, the samples were centrifuged again at 4000× *g*, and the supernatant was analyzed on a UV-VIS spectrophotometer at 560 nm.

### 4.7. Cell Surface Properties’ Characterization

The next stage of research was dedicated to the determination of changes in the cells’ surface properties occurring under the influence of contact with nitrofurazone or the products of its photocatalytic degradation. The cell suspensions were prepared, as described in Section 4.6. Then, the total membrane permeability was tested by colorimetric measurements of the uptake of crystal violet solution by microbial cells [32] by mixing 0.9 mL of microbial suspension with 0.1 mL of crystal violet solution (10 mg L^−1^). The samples were incubated for 2 h at 30 °C and centrifuged at 15,000× *g*, and the supernatant absorbance at 590 nm was measured. Analogously, the cell surface hydrophobicity was analyzed by replacing the crystal violet solution with Congo red solution (100 mg L^−1^), measuring the dye adsorption on the surface of microbial cells [33]. After the samples’ incubation and centrifugation, the supernatant absorbance was measured at 480 nm.

### 4.8. Statistical Analysis

In all analyses, at least three independent experiments were performed and the mean values were accepted as final results. The statistical significance of differences between the mean values was determined by one-way analysis of variance (ANOVA) with Tukey’s range test applied as post hoc analysis. Differences at *p* < 0.05 were considered statistically significant. The calculations were performed using Statistica v13 (StatSoft, Cracow, Poland).

## 5. Conclusions

The presented study showed that photocatalytic degradation with the tested catalysts can be a more effective way of NFZ degradation than photolysis alone. The use of the TiO_2_-P25 photocatalyst appears to be particularly beneficial. Importantly, the process is accompanied by the formation of intermediates, and the reduction in the organic carbon concentration is not at the same level as the reduction in the content of the degraded pharmaceutical. Furthermore, the experiments showed that the by-products of NFZ degradation may be toxic to bacterial cells and may affect their membranes. Therefore, it seems beneficial to combine photocatalytic degradation with biodegradation. What is more, the *A. xylosoxidans* NFZ2 strain was proved to effectively degrade both NFZ and its intermediates, which was confirmed by the substantial reduction in the organic carbon content. The obtained results indicated that the 30 min photocatalytic system using the TiO_2_-P25 photocatalyst combined with biodegradation by *A. xylosoxidans* NFZ2 allows effective mineralization (over 95%) of NFZ in contaminated water. This observation opens new and promising perspectives to reduce the threat of pharmaceutical pollution in water ecosystems.

## Figures and Tables

**Table 1:** Cell properties after exposure to sodium succinate, nitrofurazone, and its degradation products (photo, photodegradation without a catalyst; TiO_2_-P25, photocatalytic degradation with P25; Fe_3_O_4_-TiO_2_, photocatalytic degradation with Fe_3_O_4_@SiO_2_/TiO_2_); the values, which are not statistically different in data series, are marked with the same lowercase letters (“a”, “b”, etc.).

Treatment	Total Membrane Permeability (%)	Congo Red Adsorption (%)
Sodium succinate	21 ± 1 (a)	19 ± 6 (a)
Nitrofurazone	19 ± 1 (ac)	17 ± 2 (a)
Photo, 30 min	15 ± 5 (ab)	9 ± 1 (b)
Photo, 60 min	10 ± 1 (b)	12 ± 1 (c)
Fe_3_O_4_-TiO_2_, 30 min	18 ± 1 (c)	11 ± 1 (c)
Fe_3_O_4_-TiO_2_, 60 min	4 ± 1 (d)	12 ± 1 (c)
TiO_2_-P25, 30 min	5 ± 4 (de)	10 ± 2 (c)
TiO_2_-P25, 60 min	2 ± 1 (e)	14 ± 1 (d)

**Table 2:** Physical and chemical properties of the used photocatalysts.

Value	BET Surface Area (mg dm^−3^)	Anatase (%) (Size (nm))	Rutile (%) (Size (nm))	Magnetite (%) (Size (nm))
TiO_2_-P25	55	86.8 ± 0.3 (18.88 ± 0.09)	13.20 ± 0.19 (27.3 ± 0.7)	- -
Fe_3_O_4_@SiO_2_/TiO_2_	117	69.2 ± 0.8 (5.48 ± 0.05)	8.4 ± 0.5 (7.9 ± 0.4)	22.4 ± 0.8 (42.6 ± 3.3)

## Data Availability

Not applicable.

## References

[1] Cantwell M.G., Katz D.R., Sullivan J.C., Shapley D., Lipscomb J., Epstein J., Juhl A.R., Knudson C., O’Mullan G.D. (2018). Spatial Patterns of Pharmaceuticals and Wastewater Tracers in the Hudson River Estuary. Water Res..

[2] Vatovec C., Van Wagoner E., Evans C. (2017). Investigating Sources of Pharmaceutical Pollution: Survey of over-the-Counter and Prescription Medication Purchasing, Use, and Disposal Practices among University Students. J. Environ. Manag..

[3] Mandaric L., Mor J.R., Sabater S., Petrovic M. (2018). Impact of Urban Chemical Pollution on Water Quality in Small, Rural and Effluent-Dominated Mediterranean Streams and Rivers. Sci. Total Environ..

[4] Lu M.C., Chen Y.Y., Chiou M.R., Chen M.Y., Fan H.J. (2016). Occurrence and Treatment Efficiency of Pharmaceuticals in Landfill Leachates. Waste Manag..

[5] Masoner J.R., Kolpin D.W., Furlong E.T., Cozzarelli I.M., Gray J.L. (2016). Landfill Leachate as a Mirror of Today’s Disposable Society: Pharmaceuticals and Other Contaminants of Emerging Concern in Final Leachate from Landfills in the Conterminous United States. Environ. Toxicol. Chem..

[6] de Boer M.A., Hammerton M., Slootweg J.C. (2018). Uptake of Pharmaceuticals by Sorbent-Amended Struvite Fertilisers Recovered from Human Urine and Their Bioaccumulation in Tomato Fruit. Water Res..

[7] Bartelt-Hunt S.L. (2020). Fate of Veterinary Pharmaceuticals in Agroecosystems. Women in Water Quality.

[8] Wang Y., Liu J., Kang D., Wu C., Wu Y. (2017). Removal of Pharmaceuticals and Personal Care Products from Wastewater Using Algae-Based Technologies: A Review. Rev. Environ. Sci. Biotechnol..

[9] Vass M., Hruska K., Franek M. (2008). Nitrofuran Antibiotics: A Review on the Application, Prohibition and Residual Analysis. Vet. Med..

[10] Pogoda D., Janczak J., Videnova-Adrabinska V. (2016). New Polymorphs of an Old Drug: Conformational and Synthon Polymorphism of 5-Nitrofurazone. Acta Crystallogr. Sect. B Struct. Sci. Cryst. Eng. Mater..

[11] Antunes P., Machado J., Peixe L. (2006). Illegal Use of Nitrofurans in Food Animals: Contribution to Human Salmonellosis?. Clin. Microbiol. Infect..

[12] Hou S.L., Dong J., Jiang X.L., Jiao Z.H., Wang C.M., Zhao B. (2018). Interpenetration-Dependent Luminescent Probe in Indium-Organic Frameworks for Selectively Detecting Nitrofurazone in Water. Anal. Chem..

[13] Hou Y., Yuan G., Qin S., Tu L., Yan Y., Yu Z., Lin H., Chen Y., Zhu H., Song H. (2020). Photocathode Optimization and Microbial Community in the Solar-Illuminated Bio-Photoelectrochemical System for Nitrofurazone Degradation. Bioresour. Technol..

[14] Hou Y., Yuan G., Wang S., Yu Z., Qin S., Tu L., Yan Y., Chen X., Zhu H., Tang Y. (2020). Nitrofurazone Degradation in the Self-Biased Bio-Photoelectrochemical System: G-C3N4/CdS Photocathode Characterization, Degradation Performance, Mechanism and Pathways. J. Hazard. Mater..

[15] Kong D., Liang B., Yun H., Cheng H., Ma J., Cui M., Wang A., Ren N. (2015). Cathodic Degradation of Antibiotics: Characterization and Pathway Analysis. Water Res..

[16] Kong D., Yun H., Cui D., Qi M., Shao C., Cui D., Ren N., Liang B., Wang A. (2017). Response of Antimicrobial Nitrofurazone-Degrading Biocathode Communities to Different Cathode Potentials. Bioresour. Technol..

[17] De Luca M., Mas S., Ioele G., Oliverio F., Ragno G., Tauler R. (2010). Kinetic Studies of Nitrofurazone Photodegradation by Multivariate Curve Resolution Applied to UV-Spectral Data. Int. J. Pharm..

[18] Tarpani R.R.Z., Azapagic A. (2018). A Methodology for Estimating Concentrations of Pharmaceuticals and Personal Care Products (PPCPs) in Wastewater Treatment Plants and in Freshwaters. Sci. Total Environ..

[19] Angeles L.F., Mullen R.A., Huang I.J., Wilson C., Khunjar W., Sirotkin H.I., McElroy A.E., Aga D.S. (2020). Assessing Pharmaceutical Removal and Reduction in Toxicity Provided by Advanced Wastewater Treatment Systems. Environ. Sci. Water Res. Technol..

[20] Awfa D., Ateia M., Fujii M., Johnson M.S., Yoshimura C. (2018). Photodegradation of Pharmaceuticals and Personal Care Products in Water Treatment Using Carbonaceous-TiO2 Composites: A Critical Review of Recent Literature. Water Res..

[21] Mrotek E., Dudziak S., Malinowska I., Pelczarski D., Ryżyńska Z., Zielińska-Jurek A. (2020). Improved Degradation of Etodolac in the Presence of Core-Shell ZnFe_2_O_4_/SiO_2_/TiO_2_ Magnetic Photocatalyst. Sci. Total Environ..

[22] Ullah S., Ferreira-Neto E.P., Pasa A.A., Alcântara C.C.J., Acuña J.J.S., Bilmes S.A., Martínez Ricci M.L., Landers R., Fermino T.Z., Rodrigues-Filho U.P. (2015). Enhanced Photocatalytic Properties of Core@shell SiO_2_@TiO_2_ Nanoparticles. Appl. Catal. B Environ..

[23] Sinar Mashuri S.I., Ibrahim M.L., Kasim M.F., Mastuli M.S., Rashid U., Abdullah A.H., Islam A., Asikin Mijan N., Tan Y.H., Mansir N. (2020). Photocatalysis for Organic Wastewater Treatment: From the Basis to Current Challenges for Society. Catalysts.

[24] Fischer K., Kühnert M., Gläser R., Schulze A. (2015). Photocatalytic Degradation and Toxicity Evaluation of Diclofenac by Nanotubular Titanium Dioxide-PES Membrane in a Static and Continuous Setup. RSC Adv..

[25] Szabó-Bárdos E., Cafuta A., Hegedűs P., Fónagy O., Kiss G., Babić S., Škorić I., Horváth O. (2020). Photolytic and Photocatalytic Degradation of Nitrofurantoin and Its Photohydrolytic Products. J. Photochem. Photobiol. A Chem..

[26] Bergheim M., Gminski R., Spangenberg B., Debiak M., Bürkle A., Mersch-Sundermann V., Kümmerer K., Gieré R. (2015). Antibiotics and Sweeteners in the Aquatic Environment: Biodegradability, Formation of Phototransformation Products, and in Vitro Toxicity. Environ. Sci. Pollut. Res..

[27] Pacholak A., Smułek W., Zgoła-Grześkowiak A., Kaczorek E., Pacholak A., Smułek W., Zgoła-Grześkowiak A., Kaczorek E. (2019). Nitrofurantoin—Microbial Degradation and Interactions with Environmental Bacterial Strains. Int. J. Environ. Res. Public Health.

[28] Zielińska-Jurek A., Bielan Z., Wysocka I., Strychalska J., Janczarek M., Klimczuk T. (2017). Magnetic Semiconductor Photocatalysts for the Degradation of Recalcitrant Chemicals from Flow Back Water. J. Environ. Manage..

[29] Zielińska-Jurek A., Bielan Z., Dudziak S., Wolak I., Sobczak Z., Klimczuk T., Nowaczyk G., Hupka J. (2017). Design and Application of Magnetic Photocatalysts for Water Treatment. The Effect of Particle Charge on Surface Functionality. Catalysts.

[30] Wysocka I., Kowalska E., Trzciński K., Łapiński M., Nowaczyk G., Zielińska-Jurek A. (2018). UV-Vis-Induced Degradation of Phenol over Magnetic Photocatalysts Modified with Pt, Pd, Cu and Au Nanoparticles. Nanomaterials.

[31] Wang H., Cheng H., Wang F., Wei D., Wang X. (2010). An Improved 3-(4,5-Dimethylthiazol-2-Yl)-2,5-Diphenyl Tetrazolium Bromide (MTT) Reduction Assay for Evaluating the Viability of Escherichia Coli Cells. J. Microbiol. Methods.

[32] Devi K.P., Sakthivel R., Nisha S.A., Suganthy N., Pandian S.K. (2013). Eugenol Alters the Integrity of Cell Membrane and Acts against the Nosocomial Pathogen Proteus Mirabilis. Arch. Pharm. Res..

[33] Ambalam P., Kondepudi K.K., Nilsson I., Wadström T., Ljungh Å. (2012). Bile Stimulates Cell Surface Hydrophobicity, Congo Red Binding and Biofilm Formation of Lactobacillus Strains. FEMS Microbiol. Lett..

